# Supplementary figures and images for: “We have to amplify what we saw at EBOVAC” – Assessing participant perceptions, attitudes, and acceptability of an ancillary care policy in an Ebola vaccine trial in the Democratic Republic of the Congo: A mixed methods study
Source: PLoS One. 2025 Jun 24;20(6):e0325435. doi: 10.1371/journal.pone.0325435 (PMC12186984; doi:10.1371/journal.pone.0325435)

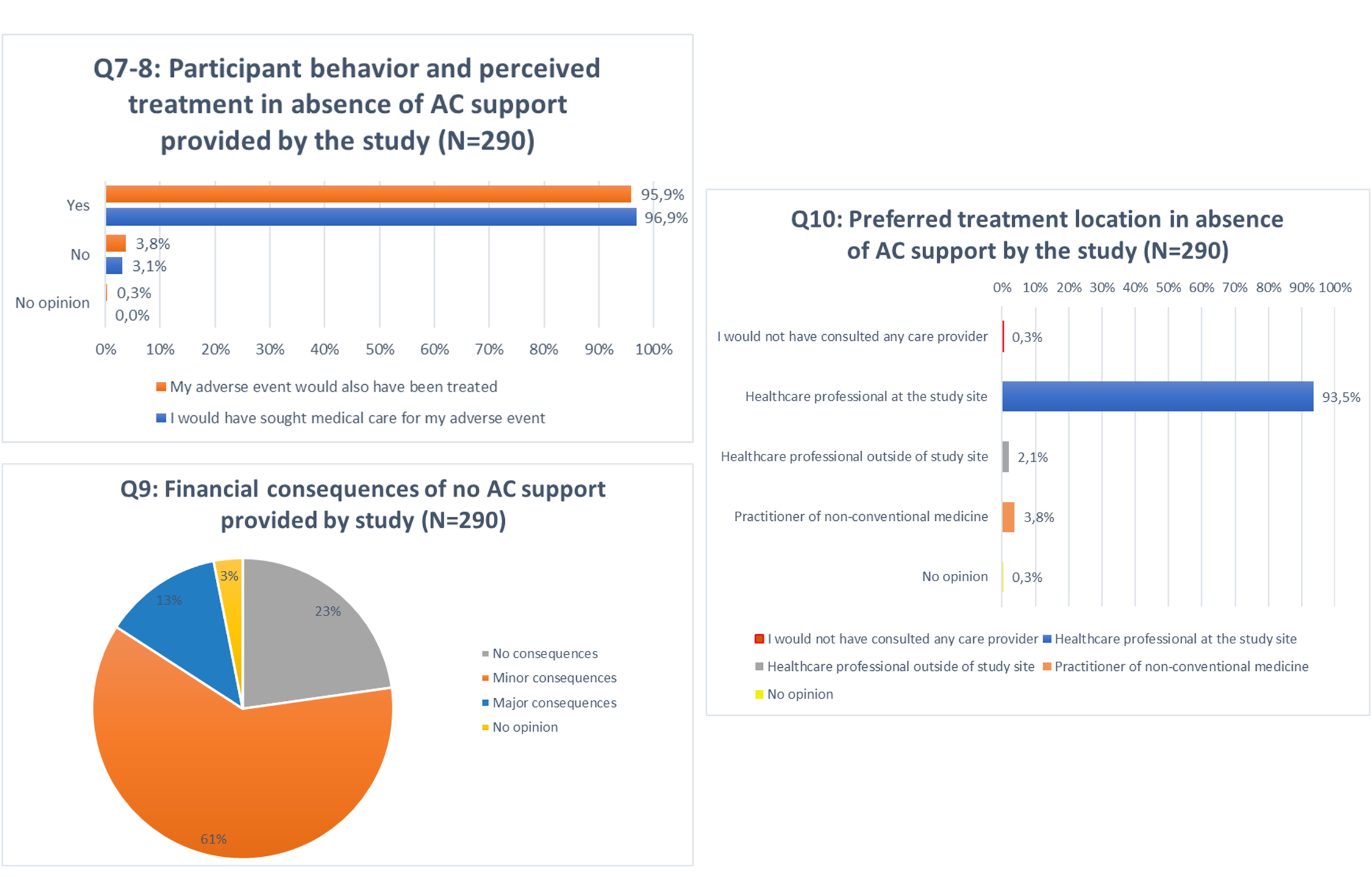

Supplement: S1 Fig — (TIF) [file pone.0325435.s001.tif]
